# Multicenter Evaluation of a Novel Surveillance Paradigm for Complications of Mechanical Ventilation

**DOI:** 10.1371/journal.pone.0018062

**Published:** 2011-03-22

**Authors:** Michael Klompas, Yosef Khan, Kenneth Kleinman, R. Scott Evans, James F. Lloyd, Kurt Stevenson, Matthew Samore, Richard Platt, for the CDC Prevention Epicenters Program

**Affiliations:** 1 Department of Population Medicine, Harvard Medical School and Harvard Pilgrim Health Care Institute, Boston, Massachusetts, United States of America; 2 Infection Control Department, Brigham and Women's Hospital, Boston, Massachusetts, United States of America; 3 Department of Medicine, The Ohio State University Medical Center, Columbus, Ohio, United States of America; 4 Department of Biomedical Informatics, University of Utah School of Medicine, Salt Lake City, Utah, United States of America; 5 Department of Medical Informatics, Intermountain Healthcare, Salt Lake City, Utah, United States of America; University of Hong Kong, Hong Kong

## Abstract

**Background:**

Ventilator-associated pneumonia (VAP) surveillance is time consuming, subjective, inaccurate, and inconsistently predicts outcomes. Shifting surveillance from pneumonia in particular to complications in general might circumvent the VAP definition's subjectivity and inaccuracy, facilitate electronic assessment, make interfacility comparisons more meaningful, and encourage broader prevention strategies. We therefore evaluated a novel surveillance paradigm for ventilator-associated complications (VAC) defined by sustained increases in patients' ventilator settings after a period of stable or decreasing support.

**Methods:**

We assessed 600 mechanically ventilated medical and surgical patients from three hospitals. Each hospital contributed 100 randomly selected patients ventilated 2–7 days and 100 patients ventilated >7 days. All patients were independently assessed for VAP and for VAC. We compared incidence-density, duration of mechanical ventilation, intensive care and hospital lengths of stay, hospital mortality, and time required for surveillance for VAP and for VAC. A subset of patients with VAP and VAC were independently reviewed by a physician to determine possible etiology.

**Results:**

Of 597 evaluable patients, 9.3% had VAP (8.8 per 1,000 ventilator days) and 23% had VAC (21.2 per 1,000 ventilator days). Compared to matched controls, both VAP and VAC prolonged days to extubation (5.8, 95% CI 4.2–8.0 and 6.0, 95% CI 5.1–7.1 respectively), days to intensive care discharge (5.7, 95% CI 4.2–7.7 and 5.0, 95% CI 4.1–5.9), and days to hospital discharge (4.7, 95% CI 2.6–7.5 and 3.0, 95% CI 2.1–4.0). VAC was associated with increased mortality (OR 2.0, 95% CI 1.3–3.2) but VAP was not (OR 1.1, 95% CI 0.5–2.4). VAC assessment was faster (mean 1.8 versus 39 minutes per patient). Both VAP and VAC events were predominantly attributable to pneumonia, pulmonary edema, ARDS, and atelectasis.

**Conclusions:**

Screening ventilator settings for VAC captures a similar set of complications to traditional VAP surveillance but is faster, more objective, and a superior predictor of outcomes.

## Introduction

Mechanically ventilated patients are at risk for a wide array of preventable pulmonary complications including pneumonia, barotrauma, fluid overload, pulmonary embolism, pneumothorax, and atelectasis. Measuring quality of care for ventilated patients, however, has focused almost exclusively on ventilator-associated pneumonia (VAP). Indeed, many groups including legislatures, accreditation agencies, and consumer organizations advocate public reporting of VAP rates with a view to benchmarking hospitals and catalyzing improvements in care.

Surveillance and public reporting of VAP, however, is problematic.[1], [2] The surveillance definition for VAP requires patients to fulfill radiologic (new and persistent infiltrate, consolidation, or cavitation), systemic (fever, abnormal white blood cell count, or delirium), and pulmonary criteria (any two of change in secretions, worsening oxygenation, rales or bronchial breath sounds, and new onset of cough or dyspnea).[3] Positive cultures of pulmonary secretions are optional. In practice, applying this definition is complicated, time consuming, and subject to substantial interobserver variability.[4], [5] There is poor correlation between clinical diagnoses of VAP and histologically confirmed infection [6]–[10] and an inconsistent correlation with patients' outcomes.[11], [12] Many patients diagnosed with VAP are found to have other complications at autopsy.[13] Requiring positive cultures adds little accuracy since endotracheal aspirates, broncholaveolar lavage, and protected specimen brush samples all have relatively poor sensitivity and specificity relative to histology.[14]–[18] Indeed, hospitals' VAP rates can vary markedly depending upon which methodology intensivists select to culture patients' pulmonary secretions.[19] Hospitals' rates also vary depending upon the prevalence of patients with common conditions that can mimic VAP in the hospitals' intensive care population.[20], [21]

The complexity, unreliability, and limited focus of VAP surveillance make VAP a poor basis for internal quality assessment or external benchmarking. Shifting the emphasis of surveillance from VAP in particular to ventilator-associated complications (VAC) in general offers many potential advantages including a) circumventing the inaccuracy of clinical signs to diagnose VAP, b) emphasizing the importance of preventing all complications of mechanical ventilation rather than pneumonia alone, and c) facilitating an objective surveillance definition that might ease the burden of data collection and make interfacility comparisons more meaningful.

We hypothesized that surveillance for increases in patients' ventilator settings after a period of stable or decreasing ventilator settings might be a good indicator of a VAC. Surveillance for increases in ventilator settings not only shifts the emphasis of surveillance to detecting multiple complications of mechanical ventilation rather than just pneumonia, but is also easier, faster, more objective, and more amenable to electronic assessment compared to the complicated weighing of clinical factors required for VAP surveillance. Furthermore, basing VAC surveillance upon increases in ventilator support sets a threshold effect for severity of complications: only patients with severe enough complications to merit a sustained increase in ventilator support are captured by this definition. It is unknown, however, whether this surveillance approach predicts patients' outcomes. Good quality measures should not only be objective and easy to gather but should also predict patients' outcomes and be able to reflect meaningful changes in quality of care.

We undertook a multicenter study to compare outcomes for patients who meet criteria for VAC compared to those who meet criteria for VAP using the traditional definition of the United States Centers for Disease Control and Prevention. In particular, we compared time for surveillance and patients' duration of mechanical ventilation, length of stay in the intensive care unit and hospital, and mortality. We also conducted a qualitative analysis of a subset of patients meeting criteria for VAC and VAP to try to determine a possible etiology for their events.

## Materials and Methods

### Setting, patients, and ethics review

This study was conducted using retrospective data from three large academic medical centers in different regions of the United States. The institutional review boards of Brigham and Women's Hospital (Boston, Massachusetts), The Ohio State University Medical Center (Columbus, Ohio), and LDS Hospital (Salt Lake City, Utah) approved the study. The review boards waived the need for informed consent since the study involved medical record review alone, all results are reported in aggregate without any personally identifiable information, and because consent was not practicable given the retrospective nature of the study and the high morbidity and mortality rate of the target population.

We assessed medical and surgical patients over age 18 on mechanical ventilation during calendar years 2006 and 2007. We had sufficient resources to review 600 patients in total. We therefore randomly selected 200 ventilated patients per hospital but enriched the sample with patients with longer durations of mechanical ventilation in order to maximize the frequency of events and hence power. In particular, each hospital randomly selected 100 patients ventilated for 2–7 days and 100 patients ventilated for >7 days. Each patient was then assessed for VAC and for VAP.

### Definitions

VAC and VAP were assessed by different reviewers blinded to each others' conclusions. VAC was manually assessed using electronically generated tables with a single line for each calendar day the patient was on mechanical ventilation. Each line listed the patient's minimum positive end expiratory pressure (PEEP) and fraction of inspired oxygen (FiO2) for that calendar day. VAC was defined as an increase in the patient's daily minimum PEEP by 2.5 cm H_2_O sustained for ≥2 days or an increase in the daily minimum FiO2 by ≥15 points sustained for ≥2 days after a minimum of 2 days of stable or decreasing daily minimum PEEPs and FiO2s respectively. Patients with persistently elevated PEEP (≥7.5 cm H2O) or FiO2 (≥70%) during the first three days of mechanical ventilation (suggesting intubation for a severe, progressive respiratory disorder) were only eligible if they subsequently stabilized and only required minimal ventilator support (PEEP ≤5 cm H2O and FiO2 ≤40%) for ≥2 days. The thresholds for defining stability prior to eligibility for VAC, the magnitude of ventilator setting increases, and the minimum duration of elevated settings were determined in advance by the investigators based on prior operational experience with a quantitative method for applying the CDC's VAP definition that incorporated changes in ventilator settings.[22] The thresholds were set at the minimum level felt to represent a meaningful change in ventilator support.

VAP was assessed by infection preventionists blinded to patients' VAC determinations. Between one and three infection preventionists in each site applied the CDC's National Healthcare Safety Network definition for ventilator-associated pneumonia to patients' charts.[3] The CDC definition requires patients to meet radiographic, systemic, and pulmonary criteria. Radiographic criteria include new or progressive and persistent infiltrate, consolidation, or cavitation. Systemic criteria include temperature >38°C, leukopenia or leukocytosis, or delirium. Pulmonary criteria require at least two of a change in sputum (new purulence, change in character, increased secretions or increased suctioning requirement); new or worsening cough, dyspnea, or tachypnea; rales or bronchial breath sounds; and/or worsening gas exchange (oxygen desaturations, increased oxygen requirements, or increased ventilator demand).

### Time for Surveillance

Two of the three study sites assessed the average time required to assess for VAP and VAC status. Both the VAC reviewer and the VAP reviewer tallied the total number of hours needed to perform surveillance for the site's 200 patients.

### Statistical analyses

Days of mechanical ventilation, days in the intensive care unit, days in the hospital, and hospital mortality were compared for patients with and without VAC and for those with and without VAP. Raw outcomes were compared using Wilcoxon signed rank test for durations and Fisher's exact test for mortality. We then did separate matched analyses for each of VAC and VAP to control for patients' pre-morbid duration of mechanical ventilation (the major risk factor for complications). All patients with VAC or VAP (“cases”) were respectively and independently matched to as many patients without VAC or VAP (“controls”) as possible. Controls' minimum duration of mechanical ventilation was at least equal to the matched cases' duration of mechanical ventilation prior to onset of VAC or VAP.[23] Patients were additionally matched on the basis of hospital, unit type (medical versus surgical), and Charlson comorbidity index. We then applied linear and logistic regression models corrected for matching in order to compare days from event to extubation, intensive care discharge, and hospital discharge in cases versus controls.[24] The day of event was defined as the day of VAC or VAP in cases, and the ventilator day on which the matched case patient developed VAC or VAP in controls. The regression models included age, sex, hospital, unit type, and Charlson comorbidity index as covariates. Time variables were log transformed in order to increase normality. The matched analyses of days from event to extubation, intensive care discharge, and hospital discharge were repeated amongst survivors only to assess whether an association between VAC and mortality might simply reflect terminal increases in ventilator support in patients with irreversible pulmonary disease. All calculations were performed using SAS version 9.2 (SAS Institute, Cary, NC).

### Qualitative analysis of VAC and VAP events

A critical care physician independently reviewed 52 patients with VAC or VAP randomly selected from one hospital. The physician was asked whether the patient suffered a significant episode of respiratory deterioration, and if so, the likely etiology. The physician reviewer was blinded to patients' VAC and VAP determinations.

## Results

During the study period, the three study hospitals provided mechanical ventilation to 11,256 patients (5,887 ventilated 0–2 days, 3,181 ventilated 2–7 days, 2,188 ventilated >7 days). Evaluable data was available for 597 patients supported for 6,347 ventilator-days. The characteristics of these patients are summarized in Table 1. Of these, 9% met the CDC definition for VAP (8.8 per 1000 ventilator days) and 23% met the definition for VAC (21.2 per 1000 ventilator days). In the two hospitals that recorded time required for surveillance, the VAP reviewer required 260 hours to assess 400 patients (mean 39 minutes per patient). The VAC reviewer required 12 hours to assess 400 patients (mean 1.8 minutes per patient).

**Table 1:** Patient Characteristics.

Patients	597
Male	328 (53%)
Age (mean)	57.5
Unit type	
Medical	299 (50%)
Surgical	286 (48%)
Mixed	12 (2%)
Comorbidities	
Coronary artery disease	117 (20%)
Cerebrovascular disease	80 (13%)
Congestive heart failure	185 (31%)
Chronic obstructive lung disease	203 (34%)
Rheumatologic disease	20 (3%)
Liver disease	142 (24%)
Diabetes	139 (23%)
Renal insufficiency	133 (22%)
Cancer	200 (34%)
Ventilator-associated pneumonia (VAP)	
Overall	55 (9%)
Ventilated ≤7 days	
Hospital A	4 (4%)
Hospital B	3 (3%)
Hospital C	0 (0%)
Ventilated >7 days	
Hospital A	28 (28%)
Hospital B	13 (13%)
Hospital C	7 (7%)
Ventilator-associated complications (VAC)	
Overall	135 (23%)
Ventilated ≤7 days	
Hospital A	9 (9%)
Hospital B	8 (8%)
Hospital C	7 (7%)
Ventilated >7 days	
Hospital A	47 (47%)
Hospital B	34 (34%)
Hospital C	30 (30%)

Patients' raw outcomes are summarized in Table 2. Both VAC and VAP were significantly associated with more days of mechanical ventilation, longer stays in the intensive care unit, and longer stays in hospital compared to VAC and VAP negative patients respectively. VAC positive patients were more likely to die than VAC negative patients but there was no difference in mortality between VAP positive versus VAP negative patients.

**Table 2:** Comparison of outcomes for ventilator-associated complication positive and negative patients and ventilator-associated pneumonia positive and negative patients.

	VAC Positive	VAC Negative	P	VAP Positive	VAP Negative	P
Number of patients	135	462	–	55	542	–
Duration of ventilation (median days)	13.0	6.0	<.001	13.5	7.0	<.001
ICU length of stay (median days)	16.3	8.0	<.001	18.0	9.0	<.001
Hospital length of stay (median days)	21.0	16.0	<.001	24.6	17.0	<.001
Hospital mortality (% of patients)	38%	23%	.001	27%	26%	1.000

Abbreviations:

VAC – ventilator associated complications; VAP – ventilator associated pneumonia.

The matched analysis is presented in Table 3. Cases were matched to between 1 and 5 control patients. All but four VAP patients and eight VAC patients were successfully matched. There were no significant differences in the age, sex, or co-morbidity profiles of cases and controls. Both VAC and VAP were significantly associated with prolonged mechanical ventilation, intensive care stay, and hospital length-of-stay compared to matched controls. However, the adjusted odds ratio of death for patients with VAC was 2.0 (95% CI 1.3–3.2, P = .003) whereas the adjusted odds ratio of death for patients VAP was 1.1 (95% CI, 0.5–2.4, P = .78).

**Table 3:** Results of linear and logistic regression models comparing patient outcomes for ventilator-associated complication or ventilator-associated pneumonia relative to matched patients without ventilator-associated complications or ventilator-associated pneumonia respectively.

	VAC Positive (95% CI)	VAC Negative (95% CI)	P	VAP Positive (95% CI)	VAP Negative (95% CI)	P
Patients matched	127	329		51	188	
Age (mean)	56.5	58.8	NS	60.4	58.0	NS
Male	56%	57%	NS	61%	56%	NS
Comorbidities						
Coronary artery disease	19%	20%	NS	10%	14%	NS
Cerebrovascular disease	9%	14%	NS	16%	16%	NS
Congestive heart failure	31%	32%	NS	18%	28%	NS
Chronic obstructive lung disease	31%	32%	NS	31%	29%	NS
Rheumatologic disease	4%	4%	NS	2%	3%	NS
Liver disease	17%	17%	NS	6%	15%	NS
Diabetes	24%	24%	NS	14%	26%	NS
Renal insufficiency	57%	42%	NS	39%	37%	NS
Cancer	49%	41%	NS	39%	36%	NS
Charlson index (mean)	2.7	2.7	NS	2.9	2.9	NS
Duration of ventilation (days)	14.7 (13.2–16.4)	9.0 (8.2–9.9)	<.001	16.9 (14.2–20.2)	11.0 (9.5–12.8)	<.001
ICU length of stay (days)	17.6 (15.7–19.6)	13.0 (11.9–14.3)	<.001	20.9 (17.7–24.7)	14.9 (13.1–17.1)	<.001
Hospital length of stay (days)	25.4 (22.7–28.4)	23.4 (21.5–25.4)	.14	30.5 (15.6–36.4)	26.8 (24.0–30.0)	.16
Days from event to extubation*	9.7 (8.4–11.2)	3.7 (3.3–4.1)	<.001	10.3 (7.9–13.4)	4.5 (3.7–5.4)	<.001
Days from event to ICU discharge*	11.8 (10.3–13.5)	6.8 (6.2–7.6)	<.001	13.2 (10.7–16.4)	7.5 (6.5–8.7)	<.001
Days from event to hospital discharge*	16.4 (14.2–18.8)	13.4 (12.1–14.8)	.01	19.7 (16.0–24.3)	15.0 (13.4–16.8)	.02
Hospital mortality (odds ratio)	2.0 (1.3–3.2)	–	.003	1.1 (0.51–2.4)	–	.78

*Date of event in cases defined as the ventilator day on which VAC or VAP began. Date of event in controls defined as the ventilator day on which the matched case patient developed VAC or VAP.

Abbreviations:

VAC – ventilator associated complications; VAP – ventilator associated pneumonia; ICU – intensive care unit.

Model adjusted for age, sex, hospital, unit type, and Charlson comorbidity index.

The matched analysis amongst survivors only is presented in Table 4. Both VAP and VAC were again significantly associated with prolonged ventilator days, intensive care days, and hospital days.

**Table 4:** Survivors only comparison of patient outcomes for patients with ventilator-associated complications or ventilator-associated pneumonia relative to matched controls.

Outcome	VAC Positive (95% CI)	VAC Negative (95% CI)	P	VAP Positive (95% CI)	VAP Negative (95% CI)	P
Duration of ventilation (days)	14.2 (12.5–16.0)	9.1 (8.2–10.0)	<.001	16.5 (13.8–19.7)	10.1 (8.8–11.8)	<.001
ICU length of stay (days)	17.4 (15.4–19.7)	13.1 (11.9–14.4)	<.001	21.6 (18.2–25.5)	14.3 (12.6–16.3)	<.001
Hospital length of stay (days)	25.4 (22.4–29.0)	23.7 (21.6–25.9)	.27	29.5 (24.3–35.7)	27.1 (24.3–30.3)	.43
Days from event to extubation*	9.0 (7.5–10.7)	3.8 (3.4–4.3)	<.001	9.8 (7.5–12.9)	3.8 (3.1–4.6)	<.001
Days from event to ICU discharge*	11.6 (9.9–13.6)	7.4 (6.6–8.2)	<.001	13.8 (11.1–17.0)	7.1 (6.2–8.1)	<.001
Days from event to hospital discharge*	18.1 (15.6–21.0)	15.1 (13.7–16.7)	.03	20.7 (16.6–25.9)	16.1 (14.3–18.2)	.05

*Date of event in cases defined as the ventilator day on which VAC or VAP began. Date of event in controls defined as the ventilator day on which the matched case patient developed VAC or VAP.

Abbreviations:

VAC – ventilator associated complications; VAP – ventilator associated pneumonia; ICU – intensive care unit.

Model adjusted for age, sex, hospital, unit type, and Charlson comorbidity index.

**Figure 1:**
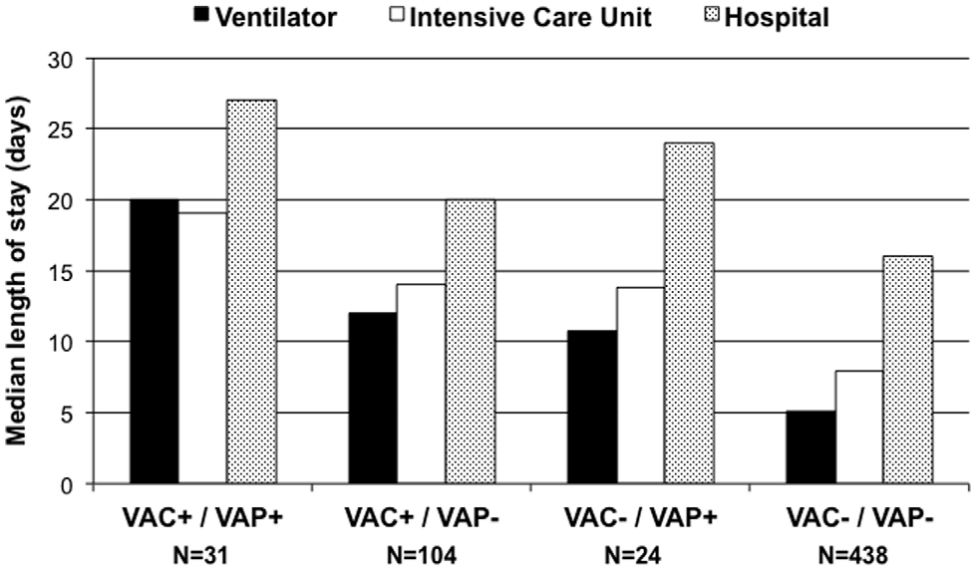
Median ventilator, intensive care unit, and hospital lengths of stay according to overlap pattern between patients with ventilator-associated complications (VAC) versus ventilator-associated pneumonia (VAP).

The frequency of overlap between patients with VAC and those with VAP is presented in Figure 1 along with median ventilator, intensive care unit, and hospital lengths of stay according to overlap pattern. The sensitivity and specificity of VAC relative to VAP were 56% (95% CI, 43–69%) and 95% (95% CI, 92–97%) respectively. Patients that met criteria for both VAC and VAP had the longest lengths of stay, those who met criteria for only one of these two conditions had similar intermediate lengths of stay, and those who were negative for both VAC and VAP had the shortest lengths of stay.

The physician's qualitative analysis of patients flagged with VAC and VAP is shown in Table 5. Similar proportions of VAC and VAP events were attributed to pneumonia (23% of VACs and 33% of VAPs), pulmonary edema (18% of VACs and 22% of VAPs), acute respiratory distress syndrome (16% of VACs and 11% of VAPs), and atelectasis (11% of VACs and 11% of VAPs).

**Table 5:** Qualitative analysis of 52 patients flagged with ventilator-associated complications or ventilator-associated pneumonia.

	Etiology of VAC (N = 44)	Etiology of VAP (N = 18)
Any pulmonary complication	26 (59%)	11 (61%)
Pneumonia	10 (23%)	6 (33%)
Pulmonary edema	8 (18%)	4 (22%)
Acute respiratory distress syndrome	7 (16%)	2 (11%)
Atelectasis	5 (11%)	2 (11%)
Mucous Plugging	1 (2%)	0
Abdominal compartment syndrome	1 (2%)	0
Pulmonary embolus	1 (2%)	0
Radiation pneumonitis	1 (2%)	0
Sepsis syndrome	1 (2%)	0
Poor pulmonary toilet	1 (2%)	0

Abbreviations:

VAC – ventilator associated complications; VAP – ventilator associated pneumonia.

## Discussion

In this multicenter retrospective study, a novel objective measure for complications of mechanical ventilation predicted patients' ventilator, intensive care, and hospital days as well as the traditional CDC surveillance definition for VAP. The novel definition for ventilator-associated complications, however, was a superior predictor of hospital mortality. Surveillance using the novel definition was faster than conventional surveillance, requiring a mean of 1.8 minutes per patient versus 39 minutes per patient for VAP. Qualitative analysis of both VAC and VAP events suggested that they were *both* predominantly attributable to similar frequencies of pneumonia, pulmonary edema, acute respiratory distress syndrome, and atelectasis.

The superior association of VAC with mortality compared to VAP might be due to the threshold effect inherent in the VAC definition. Only patients whose complications were severe enough to merit an increase in ventilator support met criteria for VAC whereas patients with stable ventilator support could still be labeled with VAP on the basis of more subjective criteria such as rales, delirium, and changes in the quality and quantity of pulmonary secretions. Indeed, about half of patients labeled with VAP did not meet criteria for VAC. By definition, these patients had stable ventilator settings despite their purported pneumonias. Mixing these stable patients with patients who do have pronounced evidence of impaired oxygenation may be the reason that VAP was not associated with increased mortality: patients with benign disease may be “diluting” the mortality signal of patients with more severe disease. As such, the relative insensitivity of VAC relative to VAP may in fact be a strength of VAC surveillance since it selects for patients with more severe and hence meaningful complications.

The association of ventilator setting increases with mortality is analogous to the partial pressure of arterial oxygen to fraction of inspired oxygen ratio (PaO_2_/FiO_2_). A sustained decrease in the PaO_2_/FiO_2_ ratio is also an independent marker for mortality in ventilated patients. Changes in ventilator settings are more suitable for continuous population surveillance than changes in PaO_2_/FiO_2_ ratios, however, since ventilator settings are available on every patient for every day of mechanical ventilation whereas PaO_2_/FiO_2_ ratios are only available when clinicians choose to obtain an arterial blood gas, typically an intermittent event.

Concern that VAC is merely a marker for the manner in which patients die on mechanical ventilation (i.e. progressive increases in ventilator support for refractory hypoxemia) is allayed by the consistent correlation between VAC and lengths of stay in the analysis of survivors alone. Likewise, VAC is unlikely to be simply a marker for severity of illness since by definition it is only triggered by a deleterious change in patients' respiratory status after a period of stable or improving respiratory status. Worsening oxygenation after a sustained period of stability or improvement is more likely to indicate a complication than progression of underlying disease.

The VAC definition can be rapidly applied either electronically or manually. The dramatically lower time required for VAC surveillance presumes the raw data is pre-organized into a linelist with each patient's daily minimum ventilator settings. Hospitals without information systems to automatically generate linelists of patients' daily minimum PEEPs and FiO2s can have nurses or respiratory therapists record these two values every 24 hours on a dedicated spreadsheet by the bedside. Spreadsheets of this nature enable infection preventionists to rapidly complete VAC surveillance by consolidating and simplifying patients' ventilator data for rapid review. Hospitals can also consider providing visual plots or statistical process control charts of daily minimum PEEPs and FiO2s for clinicians at the bedside to rapidly alert them to evolving VACs.

Many observers have questioned the validity of comparing VAP rates between hospitals as well as the clinical significance of reports of “zero” VAP rates in some hospitals. [1], [2], [25]–[27] The distribution in VAC rates between the hospitals in this study compared with the spread in VAP rates is informative. Amongst patients ventilated for 7 days or less, the observed VAP rates varied from 0 to 4% but when VAC rates were calculated, the range was only 7 to 9%, suggesting a measure that is both more uniform and able to detect complications in populations with ostensibly zero VAPs. A similar narrowing of the distribution between hospitals was observed for patients ventilated >7 days.

A potential criticism of VAC relative to VAP is that it does not indicate specific etiologies for patients' decompensations that can be used to inform future care improvement efforts. The ostensible specificity of a VAP diagnosis, however, is illusory. In this study, qualitative analysis by a critical care physician confirmed only one third of VAPs and identified many additional pneumonias missed by VAP criteria. Poor correlation between VAP clinical criteria and patients' true underlying disorders is consistent with prior investigations.[13], [28] Indeed, it is striking that similar proportions of VAC events and VAP events were attributed to same array of significant complications including pulmonary edema, acute respiratory distress syndrome, and atelectasis in addition to pneumonia. This implies that VAP surveillance both misses and mislabels many important complications. Lumping many complications together as pneumonia risks missing important alternative domains for care improvement initiatives. Labeling patients' adverse events as VACs rather than pneumonias is a more frank and therefore useful description of what can and cannot confidently be discerned by surveillance.

In addition, shifting the focus of surveillance from pneumonia alone to complications in general emphasizes the importance of preventing all complications of mechanical ventilation, not just pneumonia. Hospitals should consider treating VACs as sentinel events that catalyze a multidisciplinary, open-minded evaluation of what might have precipitated the patient's deterioration. Shifting focus from pneumonia alone to complications in general sidesteps arguments about whether or not implicated patients truly had pneumonia (a distraction that sometimes overshadows critical analyses of VAPs at present) and instead invites caregivers to try to work out what did go wrong. A sentinel analysis might conclude that the patient's deterioration was due to VAP but could just as well attribute decompensation to poor fluid management, barotrauma, thromboembolic disease, or lobar collapse secondary to mucous plugging. Ideally, open-minded analyses of complications will generate broader and more nuanced views as to what practices can be improved. Grouping VACs by suspected etiology might reveal patterns of potentially modifiable precipitants. Ultimately, this process should lead to a broader “ventilator bundle” with added measures to promote early extubation, encourage protective lung ventilation, prevent pulmonary edema, minimize blood transfusions, and better manage secretions. The Institute for Healthcare Improvement's bundle anticipates this direction: it includes thromboembolism and stress ulcer prophylaxis in addition to pneumonia specific measures such as elevating the head of the bed.[29]

There are important limitations to this work. It is a purely observational, retrospective study limited to three medical centers. VAP assignments were made by infection preventionists from chart reviews – different infection preventionists conducting prospective surveillance might have made different determinations. VAP surveillance time estimates should be treated as approximations since they measure time for retrospective chart review rather than daily, prospective, bedside assessments. The results of this investigation need to be reproduced prospectively in different settings to assure validity and generalizability.

The thresholds for stability prior to VAC eligibility, minimum changes in ventilator settings, and duration of changes merit further evaluation. In particular, the 2 day window of stable or decreasing ventilator settings prior to VAC eligibility might need to be lengthened to avoid mislabeling patients who require staggered increases in ventilator support when intubated for respiratory failure from a disease that continues to progress after intubation. Increasing the minimum thresholds for rises in ventilator settings and duration of increased ventilator support might further improve correlation between VAC and adverse outcomes.

Future changes in ventilator management strategies or the introduction of novel modes of ventilation might alter the performance or feasibility of VAC criteria. There is also some risk that clinicians may be loathe to increase patient's ventilator support, even when clinically indicated, to prevent their patient from being labeled with VAC. However, we believe the risk of this happening is low since failure to maintain patients' oxygenation in a safe zone is an egregious clinical error.

In recent years, hospitals have made admirable progress in reducing their VAP rates.[25]–[27], [30], [31] The median VAP rate in hospitals reporting to the National Safety Healthcare Network has decreased from 4.6 per 1000 ventilator-days from 1992–2004 to 2.0 per 1000 ventilator-days in 2006–2008.[32], [33] In addition, multiple hospitals have reported extended periods without any VAPs.[25]–[27] While these decreases may partly be due to the subjectivity permitted by the current VAP definition, it is clear that VAP is becoming a vanishing target upon which to focus surveillance and prevention efforts. Surveillance for VAC identifies more patients who might have suffered complications of care (almost three times as many patients met criteria for VAC compared to VAP) and therefore constitutes a broader group upon which to focus quality improvement efforts.

Most public health departments and funding agencies have shied away from compelling hospitals to report VAP rates and from making VAP a non-reimbursable event in light of the complexity and subjectivity of VAP surveillance.[1] An alternative measure is needed to promote quality assessment, benchmarking, and care improvements for ventilated patients. VAC has many features that make it a promising alternative: the definition's simplicity minimizes the extra burden upon hospital personnel to complete surveillance, its objectivity makes it less susceptible to gaming, and the close association between VAC and adverse outcomes make it a meaningful target for prevention. VAC's emphasis on complications in general rather than pneumonia per se sidesteps the inherent limitations of VAP diagnosis. This has the additional advantage of inviting thoughtful case-by-case analyses of affected patients to identify broad areas for improvements in care beyond just pneumonia prevention alone. Further study is now needed on the extent to which VAC rates can be lowered through meaningful improvements in care.
